# Functional identification of alginate lyase from the brown alga *Saccharina japonica*

**DOI:** 10.1038/s41598-019-41351-6

**Published:** 2019-03-20

**Authors:** Akira Inoue, Takao Ojima

**Affiliations:** Laboratory of Marine Biotechnology and Microbiology, Graduate School of Fisheries Sciences, Hokkaido University, 3-1-1 Minato-cho, Hakodate, Hokkaido, Japan

## Abstract

Despite the progress in massive gene analysis of brown algal species, no alginate-degrading enzyme from brown alga has been identified, impeding the understanding of alginate metabolism in brown alga. In the current study, we identified and characterized alginate lyase from *Saccharina japonica* using a protein-based approach. First, cDNA library was prepared from the *S*. *japonica* sporophyte. Expression screening was then performed; the encoding gene was identified and cloned; and the recombinant enzyme was purified and characterized. Alginate lyase production in algal tissues was evaluated by western blotting. The identified alginate lyase, SjAly (359 amino acids, with a predicted N-terminal secretion signal of 27 residues), is encoded by an open reading frame comprising seven exons. Recombinant SjAly exhibited endolytic alginate lyase activity, specifically toward stretches of consecutive β-d-mannuronic acid units. The optimum temperature, pH, and NaCl concentration were 30 °C, pH 8.0, and 100 mM, respectively. SjAly exhibited pronounced activity below 20 °C, the *S*. *japonica* growth temperature. SjAly was highly expressed in the blade but not the stipe and rhizoid. The data indicate that *S*. *japonica* possesses at least one active alginate lyase. This is the first report of a functional alginate lyase from brown alga, the major natural alginate producer.

## Introduction

Alginate is a major polysaccharide of the cell wall matrix in brown alga. It is a linear heteropolysaccharide composed of two uronic acids, β-d-mannuronic acid (M) and its C5 epimer α-l-guluronic acid (G). These units are continuously or randomly linked via 1,4-glycosidic bonds to form M-consecutive, G-consecutive, and random MG blocks^1,2^. The M and G content and ratio vary depending on various factors such as the species, plant segment, growth stage, and habitat^3–5^. The uronic acid sequence in alginate is thought to contribute to the flexibility of alga. The M:G ratio is low in the blade exposed to the waves, and high in the stipe and rhizoid that rigidly support the algal body. The MG-block is relatively the most flexible and the G-block is relatively the least flexible^6–8^. In the presence of divalent cations, such as Ca^2+^, Sr^2+^, and Ba^2+^, alginate forms a gel by a cross-linking reaction called the egg-box model^9–11^. Alginate is also biosynthesized as a biofilm component by certain terrestrial bacteria from the *Pseudomonas* and *Azotobacter* genera^12–14^. Unlike algal alginate, the bacterial alginate is often acetylated at the O2 and/or O3 positions of M residues, and acetylation affects its physicochemical and rheological properties^15–17^.

In nature, alginate lyase degrades alginate by cleaving the glycosidic bond via a β-elimination mechanism, and the enzyme is produced by alginate-assimilating organisms^18,19^. To date, considering alginate-producing organisms, functional alginate lyases have been identified in only bacteria, and not in brown algae. AlgL, an alginate lyase from *Pseudomonas aeruginosa*, has been characterized^20^ and functions in the periplasmic space during alginate biosynthesis^21–23^. However, it is not clear whether alginate-producing bacteria utilize alginate lyases for the decomposition of exopolysaccharide or alginate as a carbon source. In contrast, several alginate lyases have been well characterized in alginate-assimilating organisms such as the alga-associated bacteria *Zobellia galactanivorans*^24,25^ and *Flavobacterium* sp. strain UMI-01^26,27^. In *Z*. *galactanivorans*, alginate lyases have been shown to be essential for the decomposition of brown algal tissues for accessing carbon sources^28^. In alginate-assimilating bacteria, it has been proposed that alginate is finally metabolized to pyruvate and this process was reproduced *in vitro* using three alginate lyases, 4-deoxy-l-erythro-5-hexoseulose uronate (DEH) reductase, 2-keto-3-deoxy-D-gluconate (KDG) kinase, and 2-keto-3-deoxy-6-phosphogluconic acid (KDPG) aldolase, from *Flavobacterium* sp. strain UMI-01^29^.

Massive gene analysis of the brown alga *Ectocarpus siliculosus* indicated the presence of some proteins that share similarity with bacterial enzymes involved in alginate biosynthesis^30,31^. Hence, it was proposed that brown algae possess an alginate biosynthesis pathway similar to that of alginate-producing bacteria. If so, alginate lyase may be required during alginate biosynthesis in these organisms. However, candidate genes encoding alginate degradation enzymes have not yet been identified in brown algae^31,32^. Nevertheless, the brown algal cell wall is thought to be decomposed and regenerated *in vivo*, in response to physiological requirements such as differentiation, growth, and changes in the environment. Hence, alginate degradation is thought to be key for the understanding of brown alga biology and physiology.

On the other hand, genomic analysis of brown alga revealed unique features of algal alginate biosynthesis. Accordingly, candidate genes encoding isozymes of mannuronan C5-epimerase (MC5E) were identified. MC5E is an enzyme epimerizing the M residue to G residue on polymannuronan, and determining the sequence of M and G in alginate. Accordingly, 31 and 105 of such candidate genes were identified in the genomes of *E*. *siliculosus*^33,34^ and *Saccharina japonica*^35^, respectively. These multiple MC5Es are thought to contribute to the production of alginate molecules with a variety of M/G sequences. By contrast, *P*. *aeruginosa* and *Azotobacter vinelandii* have only one (AlgG) and seven (AlgE1–7) MC5Es, respectively^36–38^. Unlike alginates from *A*. *vinelandii* and brown alga, alginate produced by *P*. *aeruginosa* does not contain G-blocks. Interestingly, AlgE7 from *A*. *vinelandii* reportedly acts not only as a MC5E but also as an alginate lyase^39^. Therefore, it has been proposed that one or more MC5E candidates in brown alga may be involved in alginate degradation^31^. However, no such enzyme from brown alga has been described, while two recombinant MC5Es from brown alga have been investigated^34,40^.

The above led us to screen for the presence of an alginate degradation enzyme in brown alga at the protein rather than the gene level. Here, we describe the identification and characterization of a novel alginate lyase from the brown alga *S*. *japonica*. This is the first such discovery made for an alginate-biosynthesizing eukaryote and advances the understanding of alginate metabolism in the major alginate producer, the brown alga.

## Results and Discussion

### Identification of an *S*. *japonica* protein with alginate-degrading activity

Since no nucleotide or amino acid sequence information for any candidate alginate-degrading enzyme from brown alga was available before the current study, *S*. *japonica* cDNA library was constructed and expression screening performed. Out of 3280 colonies screened, one colony extract exhibited alginate degradation activity, with oligoalginate reaction products detected by thin-layer chromatography (TLC) (Supplementary Fig. S1). This suggested the presence of an alginate-degrading enzyme in brown alga. Although the sequenced cDNA clone harbored 1057 bp encoding 340 amino acids, including the termination codon, the N-terminal sequence appeared to be missing (Supplementary Fig. S2). Hence, *S*. *japonica* transcriptome database was generated and the clone sequence was used to search the database using tblastn search. One candidate sequence with a full open reading frame was identified. The candidate DNA sequence was amplified using a specific set of primers complementary to the 5′- and 3′-untranslated regions; it encoded a 359-amino acid protein, which was named SjAly (Supplementary Fig. S2). Since the N-terminal 27 residues were predicted by the SignalP 4.1 server to constitute a secretion signal peptide, we concluded that the mature SjAly consisted of 332 residues, with a molecular weight of 35,837.

**Figure 1:**
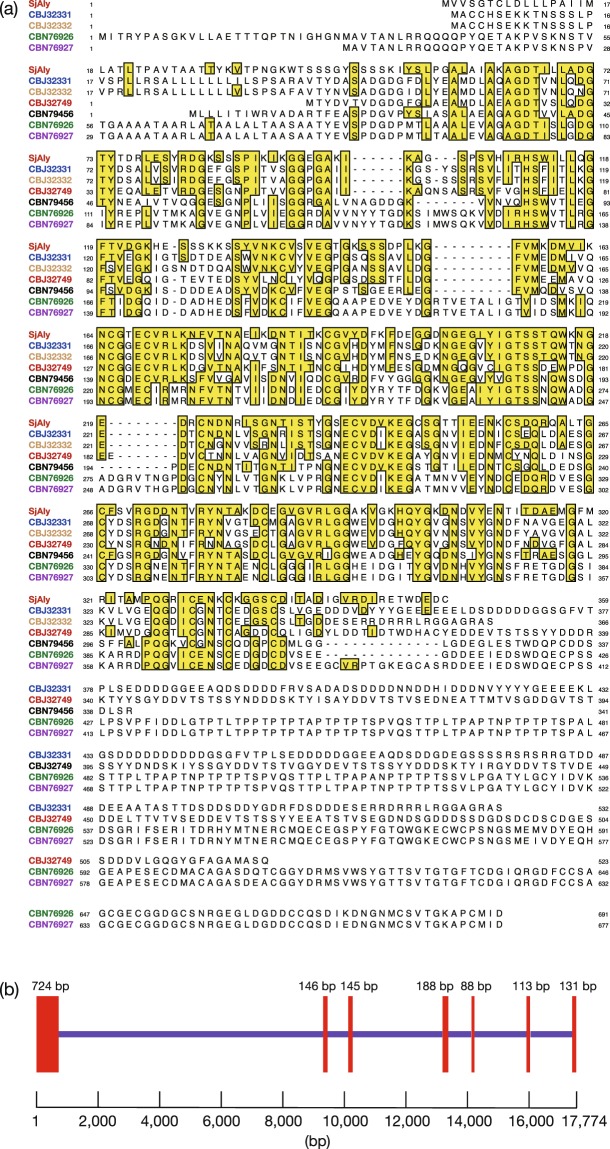
Sequence information of SjAly, the candidate protein for an alginate degradation enzyme of *Saccharina japonica*. (**a**) Comparison of amino acid sequences of SjAly and *Ectocarpus siliculosus* homologs CBJ32331, CBJ32332, CBJ32749, CBN79456, CBN76926, and CBN76927. The numbers correspond to GenBank accession numbers. Residues identical to SjAly are boxed. (**b**) Predicted genomic structure of SjAly gene. The exons and introns are shown by red boxes and blue lines, respectively.

According to the database search, SjAly shared the highest identity (48%) with a protein annotated as *E*. *siliculosus* FirrV-1-B30 precursor (nosD copper-binding protein of *Feldmannia irregularis* virus a; GenBank accession number CBN79456) whose function is unclear (Fig. 1a). Residues 1–13 of the counterpart were predicted to constitute a signal peptide for secretion. Further, SjAly shared 38–47% identity with five other proteins annotated as FirrV-1-B30 in *E*. *siliculosus*. No secretion signal peptide was predicted for three of the proteins (CBJ32749, CBN76926, and CBN76927), while four proteins (CBJ32331, CBJ32749, CBN76926, and CBN76927) harbor a C-terminal extension sequence of 171–281 residues that SjAly lacks.

To characterize the gene structure of SjAly, genomic PCR was conducted using a primer set complementary to the 5′-and 3′-untranslated regions of the SjAly gene. Unfortunately, the attempt was unsuccessful, probably because of the length of the target region or the presence of sequence(s) that are challenging to amplify (e.g., GC-rich regions). Consequently, the nucleotide sequence of SjAly gene was aligned with the genomic sequence of *S*. *japonica*^35^ available from the CoGe database (genomevolution.org/coge)^41^. A sequence identical to SjAly cDNA was identified in contig_JXRI01000040. The analysis revealed that SjAly gene consists of seven exons (Fig. 1b). All overlapping nucleotides of SjAly gene and exons were matched except that T at position 1345 of SjAly gene was replaced with C in the genome sequence, without altering the amino acid sequence. The first two and the last two nucleotides of each intron were GT and AG, respectively. This supported the notion that SjAly is an original gene product of *S*. *japonica*.

A homology model of SjAly was constructed using *Bacteroides thetaiotaomicron* rhamnogalacturonan lyase (PDB ID: 5OLQ) belonging to the polysaccharide lyase family (PL) 9 as a template, with 100% confidence (Supplementary Fig. S3). The analysis suggested that the protein contains a parallel β-helix fold. Among alginate lyases, a similar fold has been reported for *Paraglaciecola chathamensis* AlyGC from the PL-6 family^42^. Hence, amino acid sequence of SjAly was compared with those of PL-6 and PL-9 enzymes (Supplementary Figs S4 and S5). SjAly shared low identity with these enzymes: 12% with *P*. *chathamensis* AlyGC (PL-6); 13% with *Pedobacter heparinus* chondroitinase B (PL-6); 18% with *Dickeya dadantii* pectate lyase PelL (PL-9); and 13% with *B*. *thetaiotaomicron* rhamnogalacturonan lyase (PL-9). Two catalytic residues, corresponding to Lys247 and Arg268 of AlyGC^42^, are conserved in the PL-6 enzymes; the corresponding residues in SjAly are Arg221 and Val242. Of these, Arg221 is not conserved in *E*. *siliculosus* homologs, being replaced by Thr, Val, Glu, or Gly (Fig. 1a). In the PL-9 family, Asn268 and Lys273 of PelL are highly conserved among pectate lyases^43^ and are predicted to be catalytic residues; they correspond to Thr264 and Val269 of SjAly, respectively. Both residues are substituted by Ser in all *E*. *siliculosus* homologs (Fig. 1a). Considering the low conservation of SjAly residues in the vicinity of these known catalytic residues, amino acids responsible for the catalytic mechanism of SjAly may be different from those of the PL-6 and PL-9 family enzymes.

### Functional analysis of SjAly

**Figure 2:**
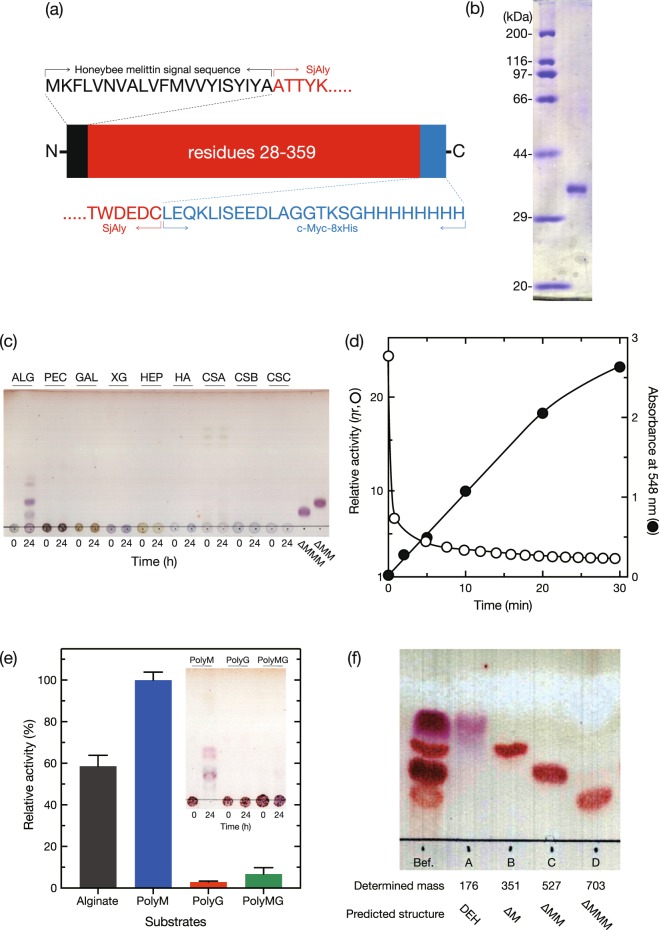
Alginate degradation by purified rSjAly. (**a**) Schematic drawing of rSjAly. (**b**) SDS-PAGE of purified rSjAly. *Left lane*, marker protein; *right lane*, purified rSjAly. Full-length gel is presented here. (**c**) TLC analysis of rSjAly reaction degradation products. *ALG*, alginate; *PEC*, pectin; *GAL*, polygalacturonic acid; *XG*, xanthan gum; *HEP*, heparin; *HA*, hyaluronic acid; *CSA*, chondroitin sulfate A; *CSB*, chondroitin sulfate B; *CSC*, chondroitin sulfate C. ΔMM and ΔMMM are unsaturated tri- and tetramannuronic acids, respectively, prepared by digestion of polyM with HULK alginate lyase. Enzyme reaction was conducted in a mixture containing 10 mM sodium phosphate (pH 8.0), 0.1 M NaCl, 0.05 mg/mL rSjAly, and 0.2% (w/v) each polysaccharide, at 20 °C for 24 h. (**d**) Evaluation of the alginate lyase activity of rSjAly. The relative viscosity (open circles) and absorbance at 548 nm after 2-thiobarbituric acid (TBA) reaction (closed circles) were determined in a mixture of 10 mM sodium phosphate (pH 8.0), 0.1 M NaCl, 0.05 mg/mL rSjAly, and 1% (w/v) sodium alginate, at 20 °C at the indicated time points. (**e**) Substrate preference of rSjAly. Enzyme reaction was conducted in a mixture containing 10 mM sodium phosphate (pH 8.0), 0.1 M NaCl, 0.02 mg/mL rSjAly, and 0.2% (w/v) each substrate, at 20 °C for 10 min. The relative activity of 100% was equivalent to 9.1 U/mg. An *inset* shows TLC analysis of rSjAly degradation products. Enzyme reaction was conducted in a mixture containing 10 mM sodium phosphate (pH 8.0), 0.1 M NaCl, 0.02 mg/mL rSjAly, and 0.2% (w/v) each substrate, at 20 °C for 24 h. (**f**) TLC analysis of fractionated polyM products of degradation by rSjAly. *Before*, the sample before column chromatography. *A*, *B*, *C*, and *D*, degradation products eluted at 50 mM, 250 mM, 350 mM, and 450 mM NH_4_Cl (Supplementary Fig. S6), respectively. Each determined mass by ESI-MS (Supplementary Fig. S7) and predicted possible structure are shown below the lane of each sample. ΔM, ΔMM, and ΔMMM are mean unsaturated di-, tri-, and tetramannuronic acids, respectively.

Recombinant enzyme (rSjAly, residues 28–332) was first expressed as a His-tagged protein in *E*. *coli*. The purified protein yield was below 100 μg from a 1-L culture, which was insufficient for biochemical characterization (data not shown). Further, as estimated by SDS-PAGE, the purity of the preparation was below 40% because of contaminating co-purified proteins. On the other hand, when rSjAly was expressed with an N-terminal HMSS-tag and C-terminal His-tag (Fig. 2a) using the baculovirus secretion expression system, at 17 °C for five days, 1.9 mg of highly pure rSjAly was successfully obtained from a 1-l culture. Molecular mass of the purified rSjAly was determined by SDS-PAGE to be approximately 35 kDa (Fig. 2b). Since the calculated mass of rSjAly without the signal peptide HMSS was 38,791 Da, the N-terminus of the protein was sequenced. The five N-terminal residues were determined to be Ala-Thr-Thr-Tyr-Lys, which was consistent with residues 28–32 of SjAly. Considering that rSjAly was purified by Ni-affinity chromatography, the protein migrated at a slightly lower position than expected on an SDS-PAGE gel. Hence, we successfully obtained rSjAly for biochemical characterization.

To investigate rSjAly substrates, the protein was incubated with several polysaccharides that are known substrates of various polysaccharide lyases (Fig. 2c). Of these, alginate was the only polysaccharide degraded by rSjAly (Fig. 2c), and TLC spots corresponding to unsaturated tri- and tetramannuronic acids were the major products. Smaller-sized decomposition products, most likely di- and monosaccharides, were also detected. Hence, alginate was the sole degradable substrate of SjAly from among the tested polysaccharides.

As shown in Fig. 2d, the relative viscosity of alginate solution was rapidly reduced by rSjAly at the early stage of the reaction, suggesting that SjAly degraded alginate in an endolytic manner. Absorbance at 548 nm of the reaction mixture after 2-thiobarbituric acid (TBA) reaction almost linearly increased in a time-dependent manner. Therefore, SjAly cleaved the glycosidic bond in alginate by a lyase mechanism.

Testing of polyM, polyG, or polyMG as substrates revealed that SjAly preferentially used polyM (Fig. 2e). At 20 °C, the specific activities with polyG (0.25 ± 0.04 U/mg) and polyMG (0.60 ± 0.11 U/mg) were lower than that with polyM (9.1 ± 0.4 U/mg). The polyM degradation products were mainly DEH, and unsaturated di-, tri-, and tetrasaccharides (Fig. 2f). Therefore, the smallest product of rSjAly-catalyzed reaction was DEH, and it was spontaneously converted from an unsaturated monosaccharide derived from alginate. On the other hand, no degradation product was detected in the polyG reaction mixture and a weak oligosaccharide signal was detected in the polyMG reaction mixture (Fig. 2e, *inset*). These observations indicated that SjAly specifically attacks M-block in an alginate molecule, producing DEH and oligosaccharides.

**Figure 3:**
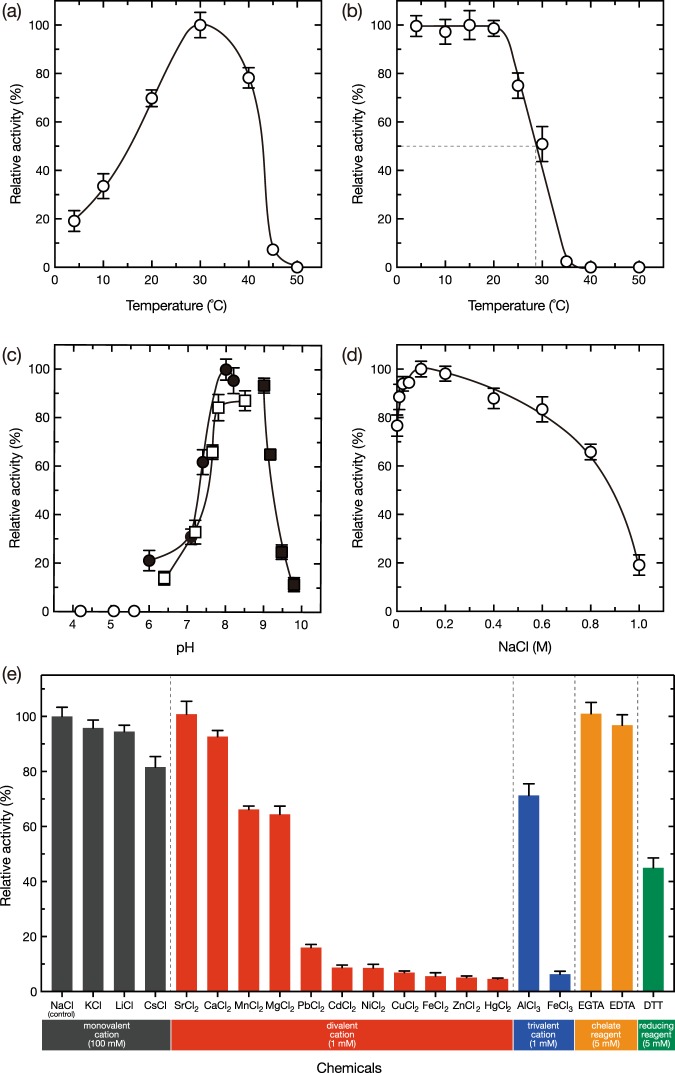
Biochemical characterization of the alginate lyase activity of rSjAly. (**a**) The effect of temperature on rSjAly activity. Enzyme reaction was conducted in a mixture containing 10 mM sodium phosphate (pH 8.0), 0.1 M NaCl, 0.02 mg/mL rSjAly, and 0.2% (w/v) polyM, at the indicated temperature for 10 min. The relative activity of 100% was equivalent to 13.8 U/mg. (**b**) The effect of temperature on rSjAly stability. A mixture containing 10 mM sodium phosphate (pH 8.0), 0.1 M NaCl, and 0.1 mg/mL rSjAly was incubated at the indicated temperature for 30 min and placed on ice for 10 min. Then, enzyme activity was assayed in a mixture containing 10 mM sodium phosphate (pH 8.0), 0.1 M NaCl, 0.02 mg/mL rSjAly, and 0.2% (w/v) polyM, at 20 °C for 10 min. The relative activity of 100% was equivalent to 8.9 U/mg. The dotted line indicates the temperature threshold for 50% loss of activity. (**c**) The effect of pH on rSjAly activity. Enzyme reaction was conducted in a mixture containing 0.1 M NaCl, 0.02 mg/mL rSjAly, 0.2% (w/v) polyM, 10 mM sodium acetate (pH 4.2–5.6, open circles), 10 mM sodium phosphate (pH 6.0–8.2, closed circles), 10 mM imidazole-HCl (pH 6.4–8.5, open squares), and 10 mM glycine-NaOH (pH 9.2–9.8, closed squares), at 20 °C for 10 min. The relative activity of 100% was equivalent to 9.1 U/mg. (**d**) The effect of NaCl or rSjAly activity. Enzyme reaction was conducted in a mixture containing 10 mM sodium phosphate (pH 8.0), 0.02 mg/mL rSjAly, 0.2% (w/v) polyM, and 0.01–1.0 M NaCl, at 20 °C for 10 min. The relative activity of 100% was equivalent to 9.4 U/mg. (**e**) The effect of various compounds on rSjAly activity. To investigate the effect of monovalent cations, a mixture of 10 mM imidazole-HCl (pH 8.0), 0.1 mg/mL rSjAly, and 100 mM each monovalent cation was incubated on ice for 1 h. The enzyme reaction was then assayed in a mixture containing 10 mM imidazole-HCl (pH 8.0), 0.02 mg/mL rSjAly, 0.2% (w/v) polyM, and 100 mM each monovalent cation, at 20 °C for 10 min. For other compounds, the incubation and assay conditions were the same except that 100 mM NaCl and the indicated concentration of each compound were used. The relative activity of 100% was equivalent to 9.5 U/mg.

Next, the effect of temperature, pH, NaCl, and various chemicals on SjAly activity was evaluated, with polyM as a substrate. The optimum temperature was determined to be 30 °C (with a specific activity of 13.8 ± 0.7 U/mg); no degradation activity was detected at 50 °C (Fig. 3a). Notably, appreciable activities were observed at 4–20 °C, a range comparable to the temperature tolerated by *S*. *japonica*. As shown in Fig. 3b, SjAly was stable after incubation at 4–20 °C for 30 min. A 50% loss of activity was observed at 28 °C after 30-min incubation, and the activity was completely abolished at 40 °C or above.

Further, rSjAly was more active under alkaline conditions than under acidic conditions (Fig. 3c). The optimum pH was close to 8, with over 80% of the maximum activity observed between pH 7.2 and 9.0. On the other hand, the lyase activity was not apparent below pH 5.6.

NaCl activated SjAly, with an optimum NaCl concentration of 100 mM (Fig. 3d). Although higher concentration of NaCl inhibited the enzyme activity, 19.1% relative activity was retained even in the presence of 1 M NaCl.

The effect of various compounds on rSjAly was also investigated (Fig. 3e). None of the tested compounds significantly activated rSjAly. Among them, Pb^2+^, Cd^2+^, Ni^2+^, Cu^2+^, Fe^2+^, Zn^2+^, Hg^2+^, and Fe^3+^ strongly inhibited the enzyme; while Mn^2+^, Mg^2+^, Al^3+^, and DTT exerted a moderate inhibitory effect. Interestingly, Ca^2+^, and the chelating reagents EDTA and EGTA did not visibly affect rSjAly activity. It has been reported that the PL-6 and PL-9 family enzymes require Ca^2+^ for activity^42,44–46^. In terms of alginate lyases, AlyGC (PL-6) requires Ca^2+^ for activity, with 0.2 mM EDTA almost completely inhibiting the enzyme and 0.2 mM Ca^2+^ fully activating the enzyme^42^. By contrast, SjAly appeared to be an alginate lyase that does not require Ca^2+^ and is not affected by divalent metal ion chelators.

The determined properties of SjAly may provide some clues for the understanding of its physiological role in *S*. *japonica*. The activity of SjAly is affected by high concentration of NaCl, suggesting that it may be active intracellularly rather than in seawater. Although brown algal alginate is thought to be biosynthesized in the Golgi apparatus^47–50^, SjAly would exhibit poor activity in such acidic organelle. Since the SjAly gene encodes a predicted signal sequence for secretion (Fig. 1a), the protein may be localized *in vivo* in the plasma membrane or at sites where alginate is secreted. Since SjAly specifically degrades consecutive M-stretches in alginate, two possible roles of the enzyme may be considered, before and/or after epimerization by MC5Es. In the former case, the protein may be responsible for degrading the nascent synthesized polymannuronan, similarly to the degradation of unexported alginate by the alginate lyase AlgL located in the periplasmic space in *Pseudomonas fluorescens*^22^. In the latter case, SjAly may contribute to the decomposition of the cell wall matrix by degrading alginate in response to such biological requirements as growth, differentiation, and changes in the environment.

### Expression of SjAly *in vivo*

**Figure 4:**
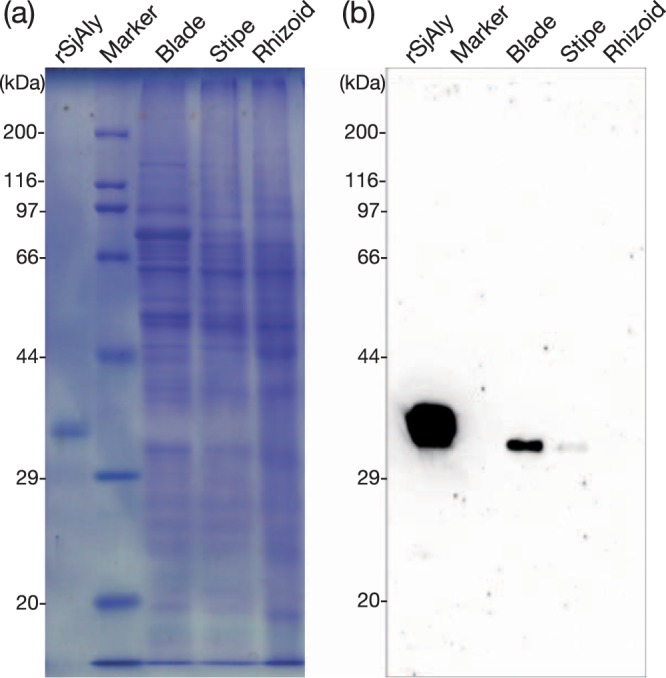
Western blot analysis of SjAly from *Saccharina japonica*. Each sample was applied to one gel in order of symmetry. After electrophoresis, the gel was cut in the middle, one stained with Coomassie Brilliant Blue and the other transferred to a nitrocellulose filter for western blotting. The primary antibody was diluted 1,000 times with 1 × TBS (20 mM Tris-HCl (pH 7.5) and 150 mM NaCl) and used, and the secondary antibody was similarly diluted 10,000 times and used. Protein bands were visualized using an ECL prime western blotting detection reagent (GE Healthcare Life Sciences, Pittsburgh, PA) and chemi-luminescence imager (EZ-capture MG, ATTO, Tokyo, Japan) in an auto exposure mode. An overexposed image is represented in Supplementary Fig. S8. (**a**) SDS-polyacrylamide gel stained using Coomassie Brilliant Blue. (**b**) Western blot analysis using anti-SjAly antibodies. *rSjAly*, purified recombinant SjAly; *Marker*, protein ladder; *Blade*, protein extract from the blade; *Stipe*, protein extract from the stipe; *Rhizoid*, protein extract from the rhizoid. Full-length gel and blot are presented here.

Next, expression of SjAly *in vivo* was investigated using a specific antibody raised against a peptide corresponding to the residues 203–222 of SjAly, in the blade, stipe, and rhizoid of *S*. *japonica* sporophyte. As shown in Fig. 4, although no signal was detected in the rhizoid extract, a protein band of approximately 33 kDa and migrating slightly quicker than rSjAly was detected in the blade and stipe extracts. The signal in the blade sample was stronger than that in the stipe sample. This suggested that the expression of SjAly was higher in the blade than in the other algal tissues. The M:G ratio in the blade is lower than that in other portions of brown alga^5,51–53^. Therefore, an M-specific alginate lyase, such as SjAly, could be involved in alginate degradation in the blade. SjAly alone does not completely decompose alginate to DEH (Fig. 2c,e). This may indicate that other alginate lyases, with properties different from those of SjAly (such as G- or MG-lyases, and an exolytic lyase) may work together if complete degradation of alginate is required. Further, the expression of G- and/or MG-lyases may be higher than that of SjAly in the stipe and rhizoid.

## Conclusions

In summary, we presented evidence that brown alga *S*. *japonica* produces at least one functional alginate lyase. This is the first report on an alginate lyase from an alginate-producing eukaryotic organism. DEH was the smallest product of alginate degradation by SjAly (Fig. 2f), which raises an interesting question about alginate metabolism in brown alga. DEH is a well-known intermediate of alginate metabolism in alginate-assimilating organisms. DEH-specific reductases, which reduce DEH to KDG using coenzyme NAD(P)H, have been characterized in *Sphingomonas* sp. strain A1^54,55^, *Z*. *galactanivorans*^24^, *Flavobacterium* sp. UMI-01^56^, *Vibrio splendidus*^57^, and abalone^58^. In alginate-assimilating bacteria, KDG is phosphorylated to KDPG by a kinase, and KDPG aldolase cleaves KDPG to pyruvate and glyceraldehyde 3-phosphate in the Entner-Doudoroff pathway^29,59,60^. If brown alga possesses a similar pathway, the enzymes involved should be investigated. The bacterial DEH-specific reductases identified to date belong to the short-chain dehydrogenase/reductase superfamily and the β-hydroxyacid dehydrogenase family, and the eukaryotic ones belongs to aldo-keto reductase superfamily. A number of candidate genes for proteins classified in these families have been identified in the sequence database of brown algae, such as *E*. *siliculosus* and *S*. *japonica*. Enzymes belonging to these families participate in the conversions of various compounds^61–64^. Although it is difficult to predict the specific substrate of each protein based on the protein primary structure, it might be possible to identify brown algal DEH-specific reductase by using the approach presented in the current study.

In the future, functional analysis and investigation of the cellular localization of alginate lyases and enzymes involved in the alginate metabolism in brown alga will allow elucidation of the biosynthesis and degradation of alginate in brown algae.

## Methods

### Materials

*S*. *japonica* sporophyte (54-cm–long) was harvested on the shore of Hakodate (Hokkaido, Japan) in June 2015. Sodium alginate (300 cps) and polygalacturonic acid were purchased from Nacalai Tesque (Kyoto, Japan). PolyM, polyG, and polyMG were prepared from alginate following the method of Gacesa and Wusteman^65^. Pectin, xanthan gum, heparin, hyaluronic acid, chondroitin sulfate B, and chondroitin sulfate C were purchased from Wako Pure Chemicals Industries (Osaka, Japan). Chondroitin sulfate A was purchased from ChromaDex (Irvine, CA). Other chemicals were purchased from Wako Pure Chemicals Industries, unless otherwise stated.

### Screening of an *S*. *japonica* cDNA expression library for alginate-degradation enzymes

Total RNA was extracted from a sporophytic thallus (0.2 g) of *S*. *japonica* using GM quicker 2 (Nippon Gene, Tokyo, Japan) and HULK alginate lyase (Nippon Gene), as described previously^66^ except that DNase I (Nippon Gene) was used instead of an attached RNase A. Then, mRNA was purified using Oligotex-dT30 Super mRNA purification kit (Takara Bio, Shiga, Japan). The cDNA was synthesized and subcloned into pAP3neo plasmid (Takara Bio) using cDNA library construction kit (Takara Bio). Recombinant pAP3neo plasmids were used as templates in PCR using PrimeStar MAX DNA polymerase (Takara Bio) and a specific primer set [primers pAP3-F, 5′-AGGTAATACACCATGATAGGTAGGGAATTCCCGGG-3′; and pAP3-R, 5′-GCGGCCGCCCTTTAGTGAGGGGATCCGGTGGAGGTG-3′ (the underlined sequences represent additional nucleotides for in-fusion cloning)] to construct the cDNA expression library. The PCR conditions were as follows: 12 cycles of 98 °C for 10 s, 55 °C for 15 s, and 72 °C for 3 min. After the removal of primers and small DNA molecules by gel filtration using a CHROMA SPIN-200 column (Takara Bio), the amplified DNA was ligated with a modified pCold vector (Takara Bio)^26^ that had been predigested with *Nco*I and *Bam*HI, using an In-Fusion HD cloning kit (Takara Bio). *Escherichia coli* BL21(DE3) (Nippon Gene) was used as the expression host; the transformants were cultured on LB agar supplemented with 50 μg/mL ampicillin at 37 °C. Each colony was cultured in 10 mL of 2×YT medium containing 50 μg/mL ampicillin at 37 °C for 12 h. Protein production was induced by incubating at 15 °C for 18 h in the presence of 0.1 mM IPTG. The cells were harvested by centrifugation at 5000 × *g* for 10 min, and then disrupted in 300 μL of BugBuster master mix (Merck, Darmstadt, Germany), according to the manufacturer’s protocol. After centrifugation at 10 000 × *g* for 10 min, 10 μL of the supernatant was mixed with 90 μL of 0.25% (w/v) sodium alginate solution containing 20 mM NaPi (pH 7.5) and 0.15 M NaCl, and the mixture was incubated at 17 °C for 24 h. The clones were screened on the basis of alginate degradation activity. Plasmid DNA extracted from the positive clone was sequenced using the BigDye Terminator v3.1 cycle sequencing kit and Applied Biosystems 3130*xl* Genetic Analyzer (both from Applied Biosystems, Foster City, CA).

### RNA sequencing of *S*. *japonica*

Total RNA from *S*. *japonica* blade was prepared as described in the preceding subsection. Transcriptome analysis was performed using the HiSeq. 3000 system (Illumina, San Diego, CA) at Hokkaido System Science (Hokkaido, Japan). Trimmed sequence reads were *de novo* assembled using the Trinity software^67,68^.

### cDNA cloning and heterologous expression

DNA encoding the full open reading frame of alginate lyase was amplified using a specific primer set (primers SjA-F1, 5′-GTGACCACCCCAACCGAAACTTTGC-3′; and SjA-R1, 5′-CCGCGGGTGACCATCAATATGCGAC-3′), and PrimeStar Max DNA polymerase, with *S*. *japonica* cDNA as a template. The PCR conditions were as follows: 2 min at 98 °C; followed by 30 cycles of 10 s denaturation at 98 °C, annealing for 15 s, and extension for 30 s. The PCR reaction products were resolved on an agarose gel, purified using an ISOSPIN agarose gel kit (Nippon Gene), treated with an A-attachment mix (Toyobo, Osaka, Japan), and subcloned into pTac-1 vector (BioDynamics, Tokyo, Japan). The analyzed nucleotide sequence is available from the DDBJ/EMBL/GenBank under the accession number LC432510.

The recombinant plasmid was used as a template in PCR [with primers SjA-F2, 5′-TCTTACATCTATGCCGCCACGACGTACAAGGTTAC-3′; and SjA-R2, 5′-AAGCTTTTGCTCGAGGCAGTCTTCATCCCACGTCT-3′ (the underlined sequences represent additional nucleotides for in-fusion cloning)], using the same reaction conditions as those described in the preceding paragraph. Amplified DNA was purified and ligated with a modified pFastBac1 vector (Thermo Fisher Scientific, Waltham, MA), in which sequences for the honeybee melittin signal peptide (HMSS; MKFLVNVALVFMVVYISYIYA, N-terminal)^69,70^ and 8×His-tag following a c-Myc-tag (EQKLISEEDL, C-terminal) were introduced^71^. Recombinant baculovirus particles were prepared according to the manufacturer’s protocol and used to infect Sf9 insect cells (Thermo Fisher Scientific; the multiplicity of infection was approximately 10). The culture was maintained in an EX-CELL 420 medium (Sigma-Aldrich, St. Louis, MO) supplemented with 5% fetal bovine serum (Thermo Fisher Scientific) in a spinner flask at 17 °C for 7 d, and then centrifuged at 3000 × *g* for 15 min. The supernatant was dialyzed against 10 mM imidazole-HCl (pH 7.4) with 0.5 M NaCl at 4 °C for 8 h. After centrifugation at 10 000 × *g* for 15 min, proteins in the supernatant were precipitated by ammonium sulfate fractionation (20–80%). The precipitates were suspended in a buffer of 10 mM imidazole-HCl (pH 7.4), 0.5 M NaCl, and 1% Triton X-100. Recombinant *S*. *japonica* alginate lyase (rSjAly) was purified using a column (1 × 3 cm) packed with His-select nickel-affinity gel (Sigma-Aldrich). The resin was washed with 20 volumes of 30 mM imidazole-HCl (pH 7.4) with 0.5 M NaCl. The protein was eluted using 250 mM imidazole-HCl (pH 7.4) with 0.5 M NaCl. After SDS-polyacrylamide gel electrophoresis (SDS-PAGE; see below), fractions containing rSjAly were collected and dialyzed against 10 mM NaPi (pH 8.0) with 0.1 M NaCl for 12 h at 4 °C. After centrifugation at 10 000 × *g* for 15 min, the samples were placed on ice and assayed within 2 d. Protein concentration was determined using Takara BCA protein assay kit (Takara Bio) and bovine serum albumin fraction V as a standard. The N-terminal amino-acid sequence was obtained using the ABI Procise 492 protein sequencer (Applied Biosystems), as per manufacturer’s guidelines.

### Alginate lyase activity assay

The reaction mixture contained 10 mM sodium phosphate (pH 8.0), 0.1 M NaCl, 0.02 mg/mL rSjAly, and 1% (w/v) sodium alginate. The decrease in viscosity of alginate solution indicative of enzyme activity was measured at 20 °C using an Ostwald-type viscometer.

Alginate lyase degradation products were analyzed by TLC using a silica gel 60 plate (Merck) unless otherwise stated. The reaction was developed in *n*-butanol, acetic acid, and water (2:1:1, v/v/v); sugars were detected by spraying the solution of 1 mg/mL 1,3-naphthalenediol, 10% (v/v) sulfuric acid, and 50% (v/v) ethanol, and then heating at 100 °C for 10 min^66^.

Alginate lyase activity of rSjAly was determined using the TBA method, as described previously^66^. One unit of activity was defined as the amount of enzyme required to liberate 1 μmol of β-formyl-pyruvic acid per min. The assay was performed three times and the data are presented as the mean ± SD.

### Fractionation of products of degradation by rSjAly and their mass spectrometry analysis

Enzyme reaction was conducted in a mixture containing 10 mM sodium phosphate (pH 8.0), 0.1 M NaCl, 0.05 mg/mL rSjAly, and 0.2% (w/v) polyM at 20 °C for 24 h. Reaction mixture (10 mL) was mixed with the same volume of chloroform and subjected to vortex mixing. The upper layer after centrifugation at 10 000 × *g* for 10 min was collected. Next, it was diluted 10-fold with distilled water and applied onto a TOYOPEARL SuperQ-650 column (1.0 × 5.0 cm) (Tosoh, Tokyo, Japan) pre-equilibrated in 10 mM NH_4_Cl. The column was washed with 100 mL of the same solution and then eluted with a step-wise gradient of 20, 50, 150, 250, 350, 450, 550, and 750 mM NH_4_Cl at a flow rate of 2.0 mL/min. Each fraction (10.0 mL) was monitored by the TBA method and peak fractions were analyzed by TLC. Samples for mass spectrometry analysis were pooled and concentrated by lyophilization as needed. Each sample was measured with an Exactive Mass Spectrometer (Thermo Fisher Scientific) with the ionization methods of electrospray ionization (ESI) at the Instrumental Analysis Division, Global Facility Center, Creative Research Institution, Hokkaido University.

### SDS-polyacrylamide gel electrophoresis

SDS-PAGE was conducted on a 10% (v/v) polyacrylamide gel. After electrophoresis, the gel was stained with 0.1% (w/v) Coomassie Brilliant Blue R-250 in 50% (v/v) methanol and 10% (v/v) acetic acid, and then destained with 5% (v/v) methanol and 7% (v/v) acetic acid. Protein molecular weight marker (broad; Takara Bio) was used as the molecular standard.

### Western blotting

Peptide N-GEGIYIGTSSTQWKNGEDRC-C was synthesized and conjugated to keyhole limpet hemocyanin by Sigma-Aldrich. Antibodies were prepared by injecting rabbits with the conjugated peptide (Sigma-Aldrich). The anti-SjAly antibody was purified by affinity chromatography using an immobilized antigen formyl-cellulofine column (Seikagaku Kogyo, Tokyo, Japan).

Proteins from the blade, stipe, and rhizoid of *S*. *japonica* sporophyte were extracted under denaturing conditions, as previously described^40^. After separation by SDS-PAGE, each protein sample was transferred by electroblotting onto a nitrocellulose membrane (Atto, Tokyo, Japan). The anti-SjAly antibody was used as the primary antibody, followed by the administration of anti-rabbit IgG secondary antibodies conjugated with horseradish peroxidase (Sigma-Aldrich). The reaction signal was detected using ECL prime western blotting detection reagent (GE Healthcare Life Sciences, Pittsburgh, PA).

### Computational analysis of SjAly

SignalP 4.1 server (www.cbs.dtu.dk/services/SignalP) was used to predict the sequence of the signal peptide for secretion^72^. Homology modeling was conducted using PHYRE2 Protein Fold Recognition Server (www.sbg.bio.ic.ac.uk/~phyre2)^73^ and visualized using CCP4^74^.

## Supplementary information

Supplementary information

## References

[1] Percival E (2007). The polysaccharides of green, red and brown seaweeds: Their basic structure, biosynthesis and function. Br. Phycol. J..

[2] Grasdalen HH-field (1983). 1H-n.m.r. spectroscopy of alginate: sequential structure and linkage conformations. Carbohydr. Res..

[3] Andresen, I.-L., Skipnes, O., Smidsrød, O., Østgaard, K. & Hemmer, P. C. In Cellulose Chemistry and Technology 48, 361–381 (AMERICAN CHEMICAL SOCIETY, 1977).

[4] Indergaard M, Skjåk-Bræk G (1987). Characteristics of alginate from *Laminaria digitata* cultivated in a high-phosphate environment. Hydrobiologia.

[5] McKee JWA, Kavalieris L, Brasch DJ, Brown MT, Melton LD (1992). Alginate content and composition of *Macrocystis pyrifera* from New Zealand. J. Appl. Phycol..

[6] Smidsrød O, Whittington SG (1969). Investigation of chemical inhomogeneity in polymers. Macromolecules.

[7] Stokke BT, Smidsrød O, Brant DA (1993). Predicted influence of monomer sequence distribution and acetylation on the extension of naturally occurring alginates. Carbohydr. Polym..

[8] Vold IMN, Kristiansen KA, Christensen BE (2006). A study of the chain stiffness and extension of alginates, *in vitro* epimerized alginates, and periodate-oxidized alginates using size-exclusion chromatography combined with light scattering and viscosity detectors. Biomacromolecules.

[9] Haug A, Smidsrød O (1967). Strontium–calcium selectivity of alginates. Nature.

[10] Haug A (1970). Selectivity of some anionic polymers for divalent metal ions. Acta Chem. Scand..

[11] Smidsrød O (1974). Molecular basis for some physical properties of alginates in the gel state. Farad. Discuss..

[12] Linker A, Jones RS (1964). A polysaccharide resembling alginic acid from a *Pseudomonas* micro-organism. Nature.

[13] Gorin PAJ, Spencer JFT (1966). Exocellular alginic acid from *Azotobacter vinelandii*. Can. J. Chem..

[14] Govan JR, Fyfe JA, Jarman TR (1981). Isolation of alginate-producing mutants of *Pseudomonas fluorescens*, *Pseudomonas putida* and *Pseudomonas mendocina*. J. Gen. Microbiol..

[15] Linker A, Jones RS (1966). A new polysaccharide resembling alginic acid isolated from Pseudomonads. J. Biol. Chem..

[16] Skjåk-Bræk G, Paoletti S, Gianferrara T (1989). Selective acetylation of mannuronic acid residues in calcium alginate gels. Carbohydr. Res..

[17] Windhues T, Borchard W (2003). Effect of acetylation on physico-chemical properties of bacterial and algal alginates in physiological sodium chloride solutions investigated with light scattering techniques. Carbohydr. Polym..

[18] Wong TY, Preston LA, Schiller NL (2000). ALGINATE LYASE: review of major sources and enzyme characteristics, structure-function analysis, biological roles, and applications. Annu. Rev. Microbiol..

[19] Ertesvåg H (2015). Alginate-modifying enzymes: biological roles and biotechnological uses. Front. Microbiol.

[20] Schiller NL, Monday SR, Boyd CM, Keen NT, Ohman DE (1993). Characterization of the *Pseudomonas aeruginosa* alginate lyase gene (algL): cloning, sequencing, and expression in *Escherichia coli*. J. Bacteriol..

[21] Albrecht MT, Schiller NL (2005). Alginate lyase (AlgL) activity is required for alginate biosynthesis in *Pseudomonas aeruginosa*. J. Bacteriol..

[22] Bakkevig K (2005). Role of the *Pseudomonas fluorescens* alginate lyase (AlgL) in clearing the periplasm of alginates not exported to the extracellular environment. J. Bacteriol..

[23] Wang Y, Moradali MF, Goudarztalejerdi A, Sims IM, Rehm BHA (2016). Biological function of a polysaccharide degrading enzyme in the periplasm. Sci. Rep..

[24] Thomas F (2012). Characterization of the first alginolytic operons in a marine bacterium: from their emergence in marine Flavobacteriia to their independent transfers to marine Proteobacteria and human gut Bacteroides. Environ. Microbiol..

[25] Thomas F (2013). Comparative characterization of two marine alginate lyases from *Zobellia galactanivorans* reveals distinct modes of action and exquisite adaptation to their natural substrate. J. Biol. Chem..

[26] Inoue A (2014). Characterization of an alginate lyase, FlAlyA, from *Flavobacterium* sp. strain UMI-01 and its expression in *Escherichia coli*. Mar. Drugs.

[27] Inoue A, Nishiyama R, Ojima T (2016). The alginate lyases FlAlyA, FlAlyB, FlAlyC, and FlAlex from *Flavobacterium* sp. UMI-01 have distinct roles in the complete degradation of alginate. Algal Res..

[28] Thomas F, Bordron P, Eveillard D, Michel G (2017). Gene expression analysis of *Zobellia galactanivorans* during the degradation of algal polysaccharides reveals both substrate-specific and shared transcriptome-wide responses. Front. Microbiol.

[29] Nishiyama R, Inoue A, Ojima T (2017). Identification of 2-keto-3-deoxy-D-gluconate kinase and 2-keto-3-deoxy-D-phosphogluconate aldolase in an alginate-assimilating bacterium, *Flavobacterium* sp. strain UMI-01. Mar. Drugs.

[30] Cock JM (2010). The *Ectocarpus* genome and the independent evolution of multicellularity in brown algae. Nature.

[31] Michel G, Tonon T, Scornet D, Cock JM, Kloareg B (2010). The cell wall polysaccharide metabolism of the brown alga *Ectocarpus siliculosus*. Insights into the evolution of extracellular matrix polysaccharides in eukaryotes. New Phytol..

[32] Moradali, M. F., Ghods, S. & Rehm, B. H. A. In Alginates and Their Biomedical Applications (eds Rehm, B. H. A. & Moradali, M. F.) 11, 1–25 (Springer Singapore, 2018).

[33] Michel G, Tonon T, Scornet D, Cock JM, Kloareg B (2010). Central and storage carbon metabolism of the brown alga *Ectocarpus siliculosus*: insights into the origin and evolution of storage carbohydrates in Eukaryotes. New Phytol..

[34] Fischl R (2016). The cell-wall active mannuronan C5-epimerases in the model brown alga *Ectocarpus*: From gene context to recombinant protein. Glycobiology.

[35] Ye N (2015). *Saccharina* genomes provide novel insight into kelp biology. Nat. Commun..

[36] Franklin MJ (1994). *Pseudomonas aeruginosa* AlgG is a polymer level alginate C5-mannuronan epimerase. J. Bacteriol..

[37] Ertesvåg H, Doseth B, Larsen B, Skjåk-Bræk G, Valla S (1994). Cloning and expression of an *Azotobacter vinelandii* mannuronan C-5-epimerase gene. J. Bacteriol..

[38] Svanem BI, Skjåk-Bræk G, Ertesvåg H, Valla S (1999). Cloning and expression of three new *Azotobacter vinelandii* genes closely related to a previously described gene family encoding mannuronan C-5-epimerases. J. Bacteriol..

[39] Svanem BI (2001). The catalytic activities of the bifunctional *Azotobacter vinelandii* mannuronan C-5-epimerase and alginate lyase AlgE7 probably originate from the same active site in the enzyme. J. Biol. Chem..

[40] Inoue A (2016). Functional heterologous expression and characterization of mannuronan C5-epimerase from the brown alga *Saccharina japonica*. Algal Res..

[41] Lyons E, Freeling M (2008). How to usefully compare homologous plant genes and chromosomes as DNA sequences. Plant J..

[42] Xu F (2017). Novel molecular insights into the catalytic mechanism of marine bacterial alginate lyase AlyGC from polysaccharide lyase family 6. J. Biol. Chem..

[43] Hassan S, Shevchik VE, Robert X, Hugouvieux-Cotte-Pattat N (2013). PelN is a new pectate lyase of *Dickeya dadantii* with unusual characteristics. J. Bacteriol..

[44] Michel G (2004). The structure of chondroitin B lyase complexed with glycosaminoglycan oligosaccharides unravels a calcium-dependent catalytic machinery. J. Biol. Chem..

[45] Jenkins J, Shevchik VE, Hugouvieux-Cotte-Pattat N, Pickersgill RW (2004). The crystal structure of pectate lyase Pel9A from *Erwinia chrysanthemi*. J. Biol. Chem..

[46] Seyedarabi A (2010). Structural insights into substrate specificity and the anti β-elimination mechanism of pectate lyase. Biochemistry.

[47] Callow ME, Coughlan SJ, Evans LV (1978). The role of Golgi bodies in polysaccharide sulphation in *Fucus zygotes*. J. Cell Sci..

[48] Schoenwaelder MEA, Wiencke C (2000). Phenolic compounds in the embryo development of several northern hemisphere fucoids. Plant Biol..

[49] Nagasato C, Motomura T (2009). Effect of latrunculin B and brefeldin A on cytokinesis in the brown alga, *Scytosiphon lomentaria* (Scytosiphonales, Phaeophyceae). J. Phycol..

[50] Nagasato C (2010). Membrane fusion process and assembly of cell wall during cytokinesis in the brown alga, *Silvetia babingtonii* (Fucales, Phaeophyceae). Planta.

[51] Venegas M, Matsuhiro B, Edding ME (1993). Alginate composition of *Lessonia trabeculata* (Phaeophyta: Laminariales) growing in exposed and sheltered habitats. Bot. Mar..

[52] Draget KI, Taylor C (2011). Chemical, physical and biological properties of alginates and their biomedical implications. Food Hydrocoll..

[53] Storz H (2009). Physicochemical features of ultra-high viscosity alginates. Carbohydr. Res..

[54] Takase R, Ochiai A, Mikami B, Hashimoto W, Murata K (2010). Molecular identification of unsaturated uronate reductase prerequisite for alginate metabolism in *Sphingomonas* sp. A1. Biochim. Biophys. Acta.

[55] Takase R, Mikami B, Kawai S, Murata K, Hashimoto W (2014). Structure-based conversion of the coenzyme requirement of a short-chain dehydrogenase/reductase involved in bacterial alginate metabolism. J. Biol. Chem..

[56] Inoue A, Nishiyama R, Mochizuki S, Ojima T (2015). Identification of a 4-deoxy-L-erythro-5-hexoseulose uronic acid reductase, FlRed, in an alginolytic bacterium *Flavobacterium* sp. strain UMI-01. Mar. Drugs.

[57] Lee EJ, Lee OK, Lee EY (2018). Identification of 4-deoxy-L-etychro-hexoseulose uronic acid reductases in an alginolytic bacterium *Vibrio splendidus* and their uses for L-lactate production in an *Escherichia coli* cell-free system. Mar. Biotechnol..

[58] Mochizuki S, Nishiyama R, Inoue A, Ojima T (2015). A novel aldo-keto reductase, HdRed, from the pacific abalone *Haliotis discus hannai*, which reduces alginate-derived 4-deoxy-l-erythro-5-hexoseulose uronic acid to 2-keto-3-deoxy-d-gluconate. J. Biol. Chem..

[59] Preiss J, Ashwell G (1962). Alginic acid metabolism in bacteria. I. Enzymatic formation of unsaturated oligosac-charides and 4-deoxy-L-erythro-5-hexoseulose uronic acid. J. Biol. Chem..

[60] Preiss J, Ashwell G (1962). Alginic acid metabolism in bacteria. II. The enzymatic reduction of 4-deoxy-L-erythro-5-hexoseulose uronic acid to 2-keto-3-deoxy-D-gluconic acid. J. Biol. Chem..

[61] Penning TM (2015). The aldo-keto reductases (AKRs): Overview. Chem. Biol. Interact..

[62] Jörnvall H (2002). Short-chain dehydrogenases/reductases (SDR). Biochemistry.

[63] Moummou H, Kallberg Y, Tonfack LB, Persson B, van der Rest B (2012). The plant short-chain dehydrogenase (SDR) superfamily: genome-wide inventory and diversification patterns. BMC Plant Biol..

[64] Njau RK, Herndon CA, Hawes JW (2001). New developments in our understanding of the β-hydroxyacid dehydrogenases. Chem. Biol. Interact..

[65] Gacesa P, Wusteman FS (1990). Plate assay for simultaneous detection of alginate lyases and determination of substrate specificity. Appl. Environ. Microbiol..

[66] Inoue A (2018). Characterization of PL-7 family alginate lyases from marine organisms and their applications. Methods Enzymol..

[67] Grabherr MG (2011). Full-length transcriptome assembly from RNA-Seq data without a reference genome. Nat. Biotechnol..

[68] Haas BJ (2013). *De novo* transcript sequence reconstruction from RNA-seq using the Trinity platform for reference generation and analysis. Nat. Protoc..

[69] Habermann E, Jentsch J (1967). Sequenzanalyse des Melittins aus den tryptischen und peptischen Spaltstücken. Biol. Chem..

[70] Vlasak R, Unger-Ullmann C, Kreil G, Frischauf A-M (1983). Nucleotide sequence of cloned cDNA coding for honeybee prepromelittin. Eur. J. Biochem..

[71] Inoue A, Mashino C, Kodama T, Ojima T (2011). Protoplast preparation from *Laminaria japonica* with recombinant alginate lyase and cellulase. Mar. Biotechnol..

[72] Petersen TN, Brunak S, Heijne, von G, Nielsen H (2011). SignalP 4.0: discriminating signal peptides from transmembrane regions. Nat. Methods.

[73] Kelley LA, Mezulis S, Yates CM, Wass MN, Sternberg MJE (2015). The Phyre2 web portal for protein modeling, prediction and analysis. Nat. Protoc..

[74] Winn MD (2011). Overview of the CCP4 suite and current developments. Acta Crystallogr. Sect. D-Biol. Crystallogr..

