# Supplementary material for: Functional identification of alginate lyase from the brown alga Saccharina japonica
Source: Sci Rep. 2019 Mar 20;9:4937. doi: 10.1038/s41598-019-41351-6 (PMC6426991; doi:10.1038/s41598-019-41351-6)
Supplement: Supplementary file 1 — Supplementary information [file 41598_2019_41351_MOESM1_ESM.pdf]

Supplementary Information

Functional identification of alginate lyase from brown alga *Saccharina japonica*

Akira Inoue* and Takao Ojima

Laboratory of Marine Biotechnology and Microbiology, Graduate School of Fisheries Sciences, Hokkaido University, 3-1-1 Minato-cho, Hakodate, Hokkaido, Japan

*Corresponding author:

E-mail: inouea21@fish.hokudai.ac.jp

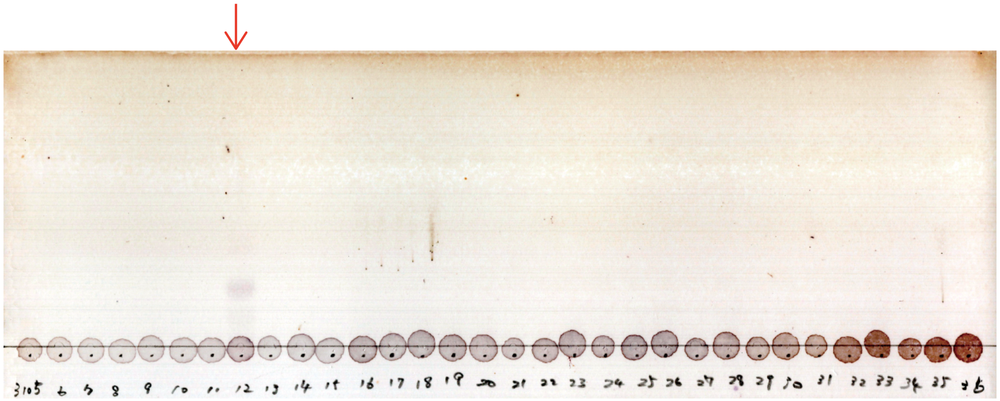

Colony number

Supplementary Fig. S1. Expression screening of a candidate gene for a protein with alginate degradation activity detected by thin-layer chromatography. Numbers correspond to the number assigned to the colony. The arrow indicates the lane recognized as alginate-degraded.

```
gtgaccaccccaaccgaaactttgcattcgaaaactccaatgtaggaaccttagtagcgc   60
ctctctctctctctctggttgtcatggtcattggcggtacggtctcgtgcctgtgcggtc  120
actgcctccgcctcccttgccatttcgcacccagacacccgtcactccgcccctctacag  180
ctccgtgccgattgaactcaccagcttcttatccgagtcaacccaattttctcggtcctc  240
tgtcatcgaacctgtggcgatccctgcgtgccgtttatctgccgtgttagcctctgcacc  300
tgcgtgcccttcccgctcagctctgcacgaagtccagggatactcatacacctacgacca  360
caacaaatcaaccATGGTGGTATCGGGAACGTGCCTTGACCTTCTCCTGCCAGCAATAAT  420
              M  V  V  S  G  T  C  L  D  L  L  L  P  A  I  I   16
TATGCTTGCCACCCTAACTCCTGCGGTTACCGCGGCCACGACGTACAAGGTTACGCCAAA  480
  M  L  A  T  L  T  P  A  V  T  A  A  T  T  Y  K  V  T  P  N   36
CGGAAAATGGACGTCTTCCTCCGGCTACAGCTCGTCGTCTAAAATTTACAGCCTCCCGGG  540
  G  K  W  T  S  S  S  G  Y  S  S  S  S  K  I  Y  S  L  P  G   56
TGCTCTGGCCATTGCCAAAGCGGGGGACACGATCCTCCTCGCAGATGGGACGTACACAGA  600
  A  L  A  I  A  K  A  G  D  T  I  L  L  A  D  G  T  Y  T  D   76
CCGCTTGGAAAGCTACAGAGACGGGAAGAGCAGCAGCCCCATCAAGATCAAGGGAGGGGA  660
  R  L  E  S  Y  R  D  G  K  S  S  S  P  I  K  I  K  G  G  E   96
GGGCGCGAAGATAAAGGCCGGGTCTCCCTCCGTGCATATCAGACATTCGTGGATACTACT  720
  G  A  K  I  K  A  G  S  P  S  V  H  I  R  H  S  W  I  L  L  116
TCAGGGCTTCACTGTGGATGGCAAGCACGAGTCTTCAAGCAAGAAGAGCTCCTACGTCAA  780
  Q  G  F  T  V  D  G  K  H  E  S  S  S  K  K  S  S  Y  V  N  136
CAAGTGCGTCAGCGTGGAAGGCACGGGGAAAAGCTCTAGCGACCCGCTCAAAGGCTTCGT  840
  K  C  V  S  V  E  G  T  G  K  S  S  S  D  P  L  K  G  F  V  156
CATGAAGGACATGGTCATCAAGAACTGCGGCACGGAGTGCGTTCGCCTGAAGAACTTCGT  900
  M  K  D  M  V  I  K  N  C  G  T  E  C  V  R  L  K  N  F  V  176
GACCAACGCCGAAATTAAGGACAACACCATCACTAAATGCGGCGTGTACGACTTCAAATT  960
  T  N  A  E  I  K  D  N  T  I  T  K  C  G  V  Y  D  F  K  F  196
TGACGAAGGAGGCGACAACGGCGAAGGCATCTATATCGGGACATCCAGTACGCAGTGGAA 1020
  D  E  G  G  D  N  G  E  G  I  Y  I  G  T  S  S  T  Q  W  K  216
AAACGGAGAGGATCGCTGTAATGACAACAGGATCAGCGGCAACACCATATCCACGTACGG 1080
  N  G  E  D  R  C  N  D  N  R  I  S  G  N  T  I  S  T  Y  G  236
CAGCGAGTGCGTCGACGTGAAGGAAGGTTGCTCCGGCACCACTATCGAGGAAAACAAGTG 1140
  S  E  C  V  D  V  K  E  G  C  S  G  T  T  I  E  E  N  K  C  256
CTCCGACCAACGGCAGGCGTTAACGGGATGCTTCAGCGTCCGGGGCGACGACAACACGGT 1200
  S  D  Q  R  Q  A  L  T  G  C  F  S  V  R  G  D  D  N  T  V  276
CAGATACAACACCGCCAAGGACTGCGAAGGCGTAGGGGTGCGGTTGGGGGGAGCGAAAGT 1260
  R  Y  N  T  A  K  D  C  E  G  V  G  V  R  L  G  G  A  K  V  296
CGGCAAGCACCAGTACGGGAAGGATAACGACGTGTACGAAAATACCATCACCGATGCGGA 1320
  G  K  H  Q  Y  G  K  D  N  D  V  Y  E  N  T  I  T  D  A  E  316
GATGGGCTTCATGAGGATCACCGCTATGCCCCAGGGGAGGATCTGCGAGAACAAATGCAA 1380
  M  G  F  M  R  I  T  A  M  P  Q  G  R  I  C  E  N  K  C  K  336
GGGCGGTTCTTGCGACATCACAGCGGATATCGGCGTTAGAGATATTCGCGAGACGTGGGA 1440
  G  G  S  C  D  I  T  A  D  I  G  V  R  D  I  R  E  T  W  D  356
TGAAGACTGCTAAacgaaaaatagggcgaattttgaaaggccgggacaaactccacccat 1500
  E  D  C  *                                                    359
atacaaacctccgcgggtgaccatcaatatgcgac                          1535
```

Supplementary Fig. S2. Nucleotide and deduced amino acid sequences of SjAly. The dotted box represents the sequence of cDNA derived from a positive clone identified by experiment shown in Supporting Information Fig. **S1**. The start and stop codons are underlined. The red arrowhead shows the cleavage site predicted by SignalP 4.1 server (www.cbs.dtu.dk/services/SignalP)[1]. Arrows indicate the positions of a primer set used for the amplification of the full open reading frame of SjAly.

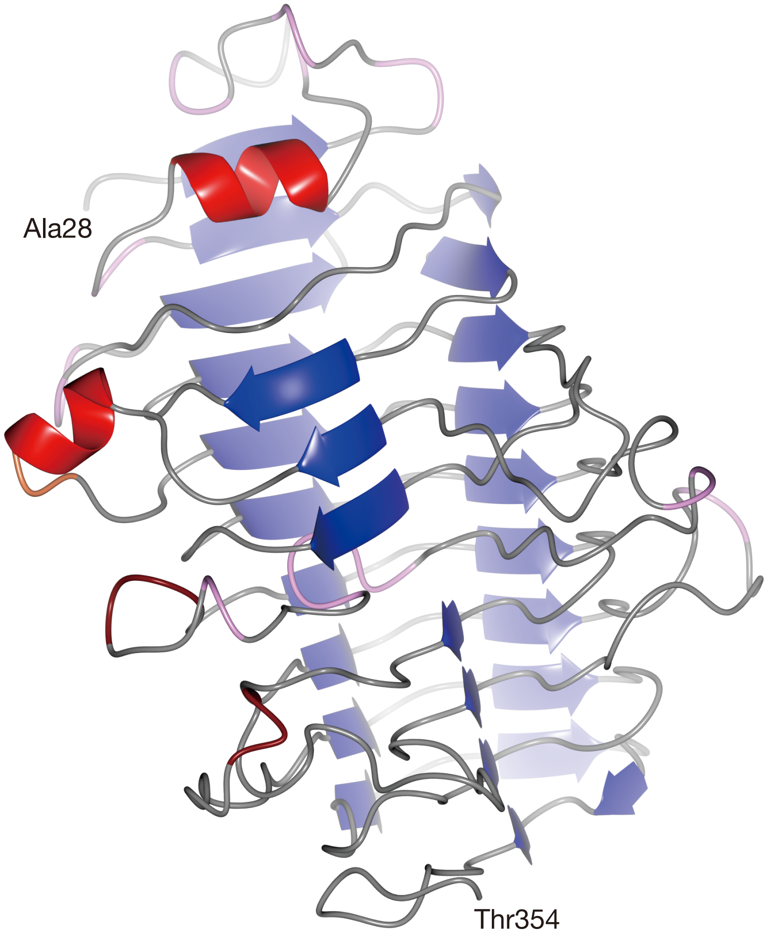
Ala28
Thr354

Supplementary Fig. S3. Homology modeling of SjAly. The structure of SjAly (residues 28–354) was predicted using the PHYRE2 program[2] and *Bacteroides thetaiotaomicron* rhamnogalacturonan lyase (PDB ID: 5OLQ) with 100% confidence.

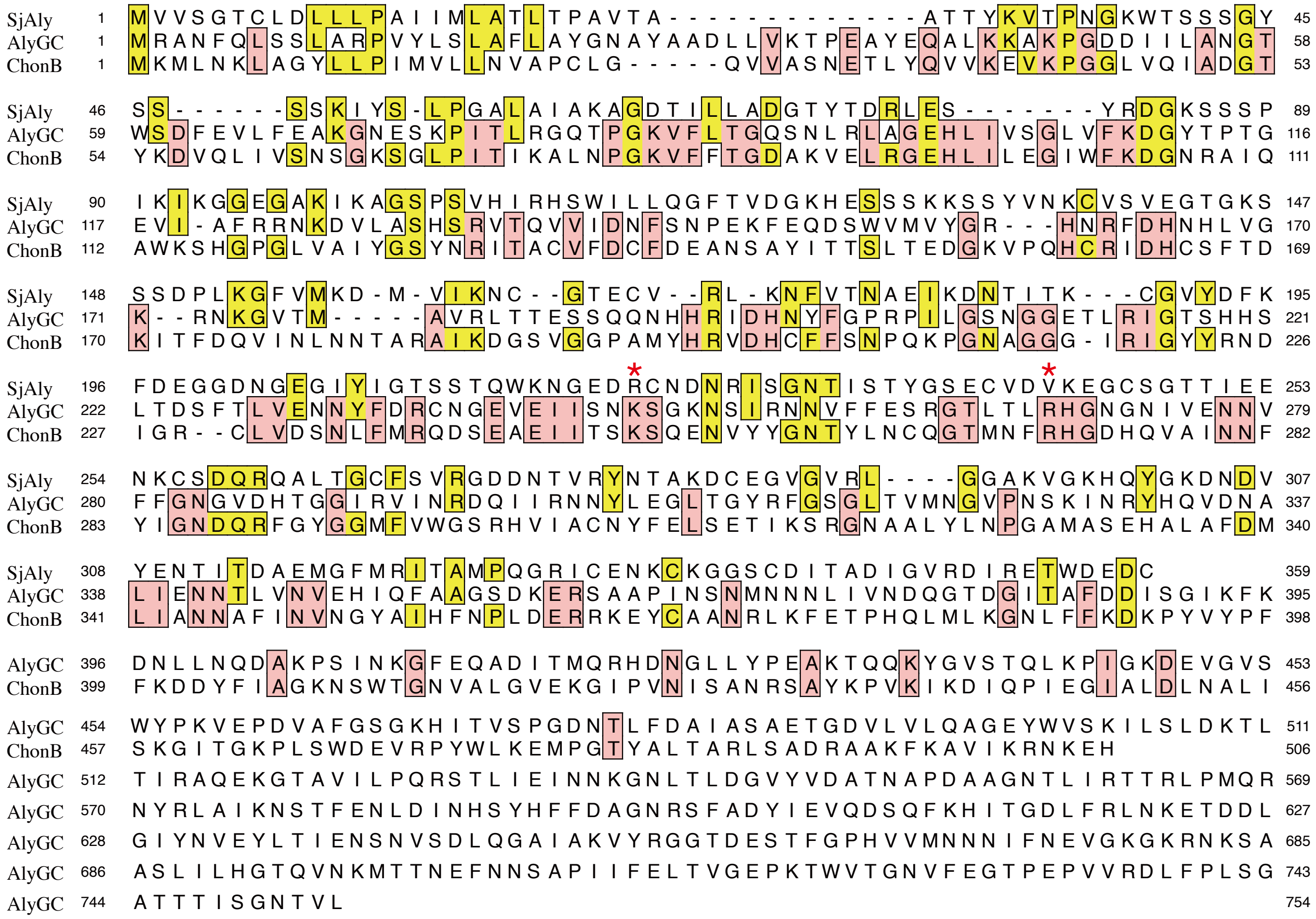

SjAly 1 MVVSGTCLDLLLPAIIMLATLTPAVTA-------------ATTYKVTPNGKWTSSSGY 45
AlyGC 1 MRANFQLSSLARPVYLSLAFLAYGNAYAADLLVKTPEAYEQALKKAKPGDDIILANGT 58
ChonB 1 MKMLNKLAGYLLPIMVLLNVAPCLG-----QVVASNETLYQVVKEVKPGGLVQIADGT 53
SjAly 46 SS------SSKIYS-LPGALAIAKAGDTILLADGTYTDRLES-------YRDGKSSSP 89
AlyGC 59 WSDFEVLFEAKGNESKPITLRGQTPGKVFLTGQSNLRLAGEHLIVSGLVFKDGYTPTG 116
ChonB 54 YKDVQLIVSNSGKSGLPITIKALNPGKVFFTGDAKVELRGEHLILEGIWFKDGNRAIQ 111
SjAly 90 IKIKGGEGAKIKAGSPSVHIRHSWILLQGFTVDGKHESSSKKSSYVNKCVSVEGTGKS 147
AlyGC 117 EVI-AFRRNKDVLASHSRVTQVVIDNFSNPEKFEQDSWVMVYGR---HNRFDHNHLVG 170
ChonB 112 AWKSHGPGLVAIYGSYNRITACVFDCFDEANSAYITTSLTEDGKVPQHCRIDHCSFTD 169
SjAly 148 SSDPLKGFVMKD-M-VIKNC--GTECV--RL-KNFVTNAEIKDNTITK---CGVYDFK 195
AlyGC 171 K--RNKGVTM-----AVRLTTESSQQNHHRIDHNYFGPRPILGSNGGETLRIGTSHHS 221
ChonB 170 KITFDQVINLNNTARAIKDGSVGGPAMYHRVDHCFFSNPQKPGNAGGG-IRIGYYRND 226
SjAly 196 FDEGGDNGEGIYIGTSSTQWKNGEDRCNDNRISGNTISTYGSECVDVKEGCSGTTIEE 253
AlyGC 222 LTDSFTLVENNYFDRCNGEVEIISNKSGKNSIRNNVFFESRGTLTLRHGNGNIVENNV 279
ChonB 227 IGR--CLVDSNLFMRQDSEAEIITSKSQENVYYGNTYLNCQGTMNFRHGDHQVAINNF 282
SjAly 254 NKCSDQRQALTGCFSVRGDDNTVRYNTAKDCEGVGVRL----GGAKVGKHQYGKDNDV 307
AlyGC 280 FFGNGVDHTGGIRVINRDQIIRNNYLEGLTGYRFGSGLTVMNGVPNSKINRYHQVDNA 337
ChonB 283 YIGNDQRFGYGGMFVWGSRHVIACNYFELSETIKSRGNAALYLNPGAMASEHALAFDM 340
SjAly 308 YENTITDAEMGFMRITAMPQGRICENKCKGGSCDITADIGVRDIRETWDEDC 359
AlyGC 338 LIENNTLVNVEHIQFAAGSDKERSAAPINSNMNNNLIVNDQGTDGITAFDDISGIKFK 395
ChonB 341 LIANNAFINVNGYAIHFNPLDERRKEYCAANRLKFETPHQLMLKGNLFFKDKPYVYPF 398
AlyGC 396 DNLLNQDAKPSINKGFEQADITMQRHDNGLLYPEAKTQQKYGVSTQLKPIGKDEVGVS 453
ChonB 399 FKDDYFIAGKNSWTGNVALGVEKGIPVNISANRSAYKPVKIKDIQPIEGIALDLNALI 456
AlyGC 454 WYPKVEPDVAFGSGKHITVSPGDNTLFDAIASAETGDVLVLQAGEYWVSKILSLDKTL 511
ChonB 457 SKGITGKPLSWDEVRPYWLKEMPGTYALTARLSADRAAKFKAVIKRNKEH 506
AlyGC 512 TIRAQEKGTAVILPQRSTLIEINNKGNLTLDGVYVDATNAPDAAGNTLIRTTRLPMQR 569
AlyGC 570 NYRLAIKNSTFENLDINHSYHFFDAGNRSFADYIEVQDSQFKHITGDLFRLNKETDDL 627
AlyGC 628 GIYNVEYLTIENSNVSDLQGAIAKVYRGGTDESTFGPHVVMNNNIFNEVGKGKRNKSA 685
AlyGC 686 ASLILHGTQVNKMTTNEFNNSAPIIFELTVGEPKTWVTGNVFEGTPEPVVRDLFPLSG 743
AlyGC 744 ATTTISGNTVL 754

Supplementary Fig. S4. Comparison of SjAly amino acid sequence with those of PL-6 family enzymes. *SjAly*, *Saccharina japonica* alginate degradation enzyme candidate (present study); *AlyGC*, *Glaciecola chathamensis* alginate lyase AlyGC (NCBI reference sequence number WP_007984897)[3]; *ChonB*, *Pedobacter heparinus* chondroitinase B (GenBank accession number ACU03011)[4]. Residues identical to SjAly, and residues conserved only between AlyGC and ChonB are shown by yellow and pink boxes, respectively. The proposed two catalytic residues of AlyGC[3], Lys247, and Arg268, are indicated by asterisks.

```
                                                                                  *          *
SjAly      1  MVVSGTCLDLLLPAIIMLATLTPAVTA-----------ATTYKVTPNGKWTSSS   43
PelL       1  MKYLNCFISTGLAAFFLVNSTSVLAADCSSDLTSGISTKRIYYVAPNG--NSSN   52
RG lyase   1  MKDITKITYLLLGLMLSVPLAAQK----------------TYYMDPEG--SDSN   36

SjAly     44  GYSSSSKIYSLPGALAIAKAGDTILLADGTYTDRLESYR---------------   82
PelL      53  NGSSFNAPMSFSAAMAAVNPGELILLKPGTYTIPYTQGKGNTIT---------F   97
RG lyase  37  PGTSDKPFATLVKVQEVVVAGDVVYINPGTYVVPANQVPMTTTNSGLYHCVFHM   90

SjAly     83  --DGKSSSPIKIKGGEGAKIKAGSPSVHIRHSWILL-QGFTVDG-------KHE  126
PelL      98  NKSGKDGAPIYVAAAN--CGRAVFDFSFPDSQWVQASYGFYVTGDYWYFKGVEV  149
RG lyase  91  NKSGEAGKPISYLANPNKQGRPIFDLSQVKP-KDQRITVFYVTGSNLYLKGFDV  143

SjAly    127  SSSKKSSYVNKCVSVEGTGKSSSDPLKGFVMKDMVIKNCGTECVRLKNFVTNAE  180
PelL     150  TRAGYQGAYVIGSHNTFENTAFHHNRNTGLEINN---------GGSYNTVINSD  194
RG lyase 144  IGTQVTITGHTQSECFRIVKGANNNKFEDLRTHDGMAIGFYLLGGSNNHILNCD  197

SjAly    181  IKDNTITKCGVYDFKFDEGGDNGEGIYIGTSSTQWKNGEDRCNDNRISGNTIST  234
PelL     195  AYRNYDPKKNGS---MADGFGPK-------QKQGPGNRFVGCRAWENSDDGFDL  238
RG lyase 198  AYNNYDSVSEGGKGGNVDGFGGHINSSSVGEGKGTGNVFEGCRAWYNSDDGFDL  251

                                           *    *
SjAly    235  YGSECVDVKEGCSGTTIEENKCSDQRQALTGCFSVRGDD----------NTVRY  278
PelL     239  FDSPQKVVIENSWAFRNGINYWNDSAFAGNGNGFKLGGN----------QAVGN  282
RG lyase 252  INCFEAVKIINCWSFLNGYKPGTKEVAGDGTGFKAGGYGMAADKLPAIPSVIPQ  305

SjAly    279  NTAKDCEGVGVRLGGAKVGKHQYGKDNDVYENTITDAEMGFMRIT---AMP---  326
PelL     283  HRITRSVAFGNVSKGFDQNNNAGGVTVINNTSYKNGINYGFGSNVQ--------  328
RG lyase 306  HEVRNSLAYYNRLRGFYANHHLGGIIFESNTAVNSGENYNMTNRESPLALPPTD  359

SjAly    327  -QGRICENK----CKGGSCDITADIGVRDIRETWDEDC                 359
PelL     329  -SGQKHYFRNNVSLSAS----VTVSNADAKSNSWDTGPAASAS----DFVSLDT  373
RG lyase 360  VNGYDHMVKNNLSLVTRSGSKHIVMVNRAKSEVSNNSFDGSEEVIETDFISLEE  413

PelL     374  SLATVSRDNDGTLPETSLFRLSANSKLINAGTKESNISYSGSAPDLGAFERN    425
RG lyase 414  AELMRDRKPNGDLPDVNFGKLTTDAELRFWGMGCFATGEPTDLDFGWLKKPTIV  467

RG lyase 468  VVGSKASVVGPEAASFTKMYVIVDGEETTEFDKNSIDLSDFSGVLEVKAVIEDA  521

RG lyase 522  NGNITKSIALKFKR                                          535
```

Supplementary Fig. S5. Comparison of SjAly amino acid sequence with those of PL-9 family enzymes. *SjAly*, *Saccharina japonica* alginate degradation enzyme candidate (present study); *PelL*, *Dickeya dadantii* pectate lyase L (GenBank accession number ADM99100)[5]; *RG lyase*, *Bacteroides thetaiotaomicron* rhamnogalacturonan lyase (GenBank accession number AAO79275). Residues identical to SjAly, and residues conserved only between PelN and RG lyase are shown by yellow and pink boxes, respectively. The proposed two catalytic residues of PelL[6], Asn268 and Lys273, are indicated by asterisks.

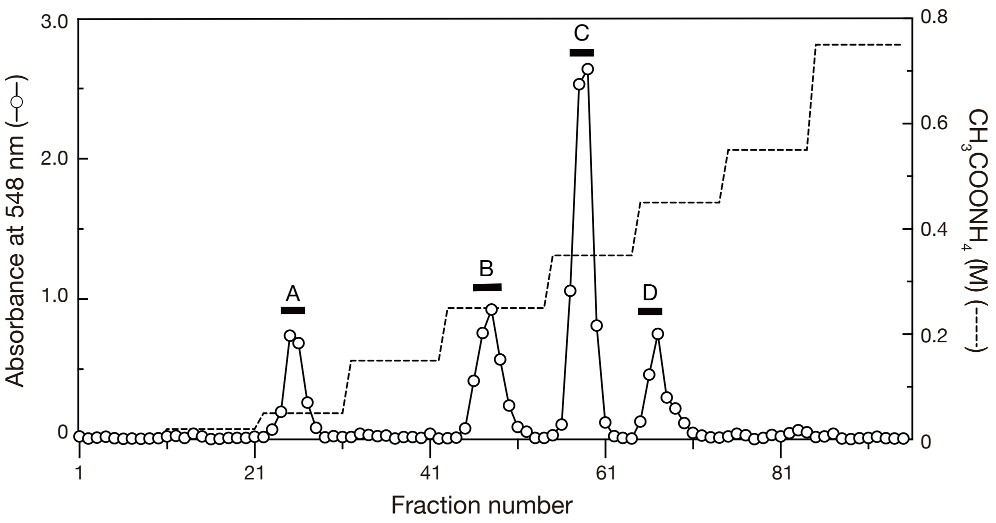

3.0
2.0
1.0
0
Absorbance at 548 nm (–○–)
A
B
C
D
1
21
41
61
81
Fraction number
0.8
0.6
0.4
0.2
0
CH_3COONH_4 (M) (----)

Supplementary Fig. S6. Ion-exchange chromatography of products of degradation by rSjAly. Fractions A, B, C, and D indicated by horizontal lines were pooled, respectively. Fractions B and D were concentrated by lyophilization, respectively. Each fraction was analyzed by TLC on Silicagel 70 F_{254} TLC Plate-Wako (Wako Pure Chemicals Industries) (Fig. 2f).

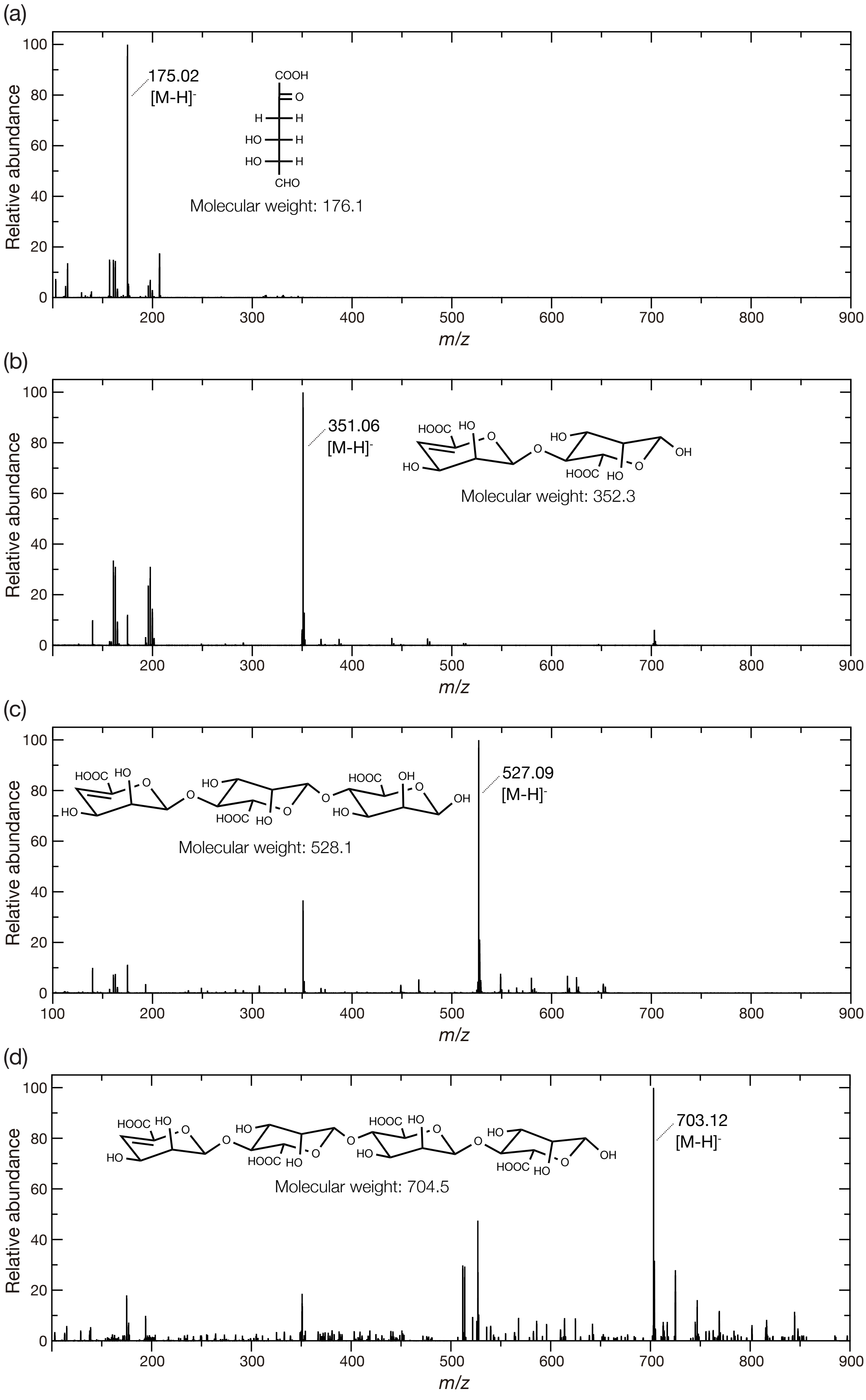
(a)
175.02
[M-H]-
COOH
O
H
H
HO
H
HO
H
CHO
Molecular weight: 176.1
Relative abundance
m/z
(b)
351.06
[M-H]-
HOOC
HO
O
O
HO
OH
HO
HOOC
HO
O
Molecular weight: 352.3
Relative abundance
m/z
(c)
527.09
[M-H]-
HOOC
HO
O
O
HO
HOOC
HO
O
O
HOOC
OH
O
HO
OH
Molecular weight: 528.1
Relative abundance
m/z
(d)
703.12
[M-H]-
HOOC
HO
O
O
HO
HOOC
HO
O
O
HOOC
HO
O
HO
O
HO
HOOC
HO
O
OH
Molecular weight: 704.5
Relative abundance
m/z

Supplementary Fig. S7. Mass spectrometry analysis of products of degradation by rSjAly. Panels a, b, c, and d represent the negative ion ESI mass spectra of fractions A, B, C, and D in Figure S6, respectively. Possible structure predicted from each determined mass is shown as an *inset*.

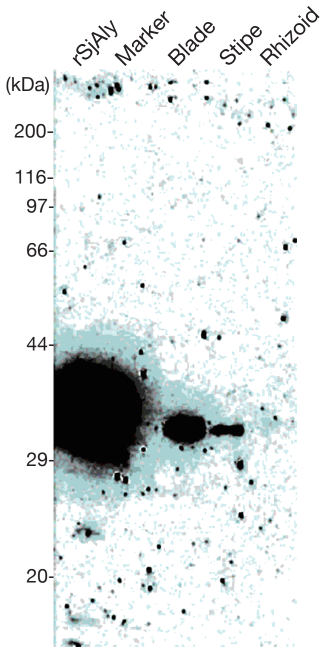
rSjAly
Marker
Blade
Stipe
Rhizoid
(kDa)
200-
116-
97-
66-
44-
29-
20-

Supplementary Fig. S8. An overexposed image of Fig 4b.

References

1. Petersen, T. N., Brunak, S., Heijne, von, G. & Nielsen, H. SignalP 4.0: discriminating signal peptides from transmembrane regions. *Nat. Methods* **8,** 785–786 (2011).
2. Kelley, L. A., Mezulis, S., Yates, C. M., Wass, M. N. & Sternberg, M. J. E. The Phyre2 web portal for protein modeling, prediction and analysis. *Nat. Protoc.* **10,** 845–858 (2015).
3. Xu, F. *et al.* Novel molecular insights into the catalytic mechanism of marine bacterial alginate lyase AlyGC from polysaccharide lyase family 6. *J. Biol. Chem.* **292,** 4457–4468 (2017).
4. Huang, W. *et al.* Crystal structure of chondroitinase B from *Flavobacterium heparinum* and its complex with a disaccharide product at 1.7 A resolution. *J. Mol. Biol.* **294,** 1257–1269 (1999).
5. Lojkowska, E., Masclaux, C., Boccara, M., Robert-Baudouy, J. & Hugouvieux-Cotte-Pattat, N. Characterization of the pelL gene encoding a novel pectate lyase of *Erwinia chrysanthemi* 3937. *Mol. Microbiol.* **16,** 1183–1195 (1995).
6. Hassan, S., Shevchik, V. E., Robert, X. & Hugouvieux-Cotte-Pattat, N. PelN is a new pectate lyase of *Dickeya dadantii* with unusual characteristics. *J. Bacteriol.* **195,** 2197–2206 (2013).
